# Mapping Protein Diffusion from the Plasma Membrane to the Nucleus: Insights from Fluorescence Correlation Spectroscopy

**DOI:** 10.3390/biom16081143

**Published:** 2026-08-06

**Authors:** Zahra Nadia Saadatmand, Nazanin Ghaderinejad, Elizabeth Hinde

**Affiliations:** 1 La Trobe Institute for Molecular Science, La Trobe University, Melbourne, VIC 3086, Australia; 2 School of Physics, University of Melbourne, Parkville, VIC 3010, Australia

**Keywords:** fluorescence correlation spectroscopy, protein dynamics, membrane diffusion, nucleocytoplasmic transport, DNA target search

## Abstract

Fluorescence correlation spectroscopy (FCS) measures spontaneous temporal fluorescence fluctuations within a femtolitre observation volume to extract, with single-molecule sensitivity, the concentration, mobility, oligomeric state, and interactions of proteins in living cells. This review traces how FCS and its spatiotemporal derivatives, implemented on different types of optical microscopes, have mapped protein trafficking across the three physically distinct environments a protein must navigate from the cell surface to its genomic targets. At the plasma membrane, FCS resolves nanodomains with millisecond confinement times and distinguishes cytoskeletal corralling from cholesterol-dependent trapping through the FCS diffusion law, revealing how receptor signalling is organised below the diffraction limit. In the cytoplasm, FCS quantifies how macromolecular crowding slows protein diffusion by a factor of 3–4 relative to water, drives anomalous sub-diffusion, and coexists with directed transport, while resolving the markedly slower dynamics of liquid–liquid phase-separated condensates. In the nucleus, FCS-derived pair correlation and brightness analyses show that chromatin acts as a size-selective filter where an inert protein dimer can take more than 10-fold longer than its monomer to traverse the same nuclear distance, and that this oligomeric-state-dependent gating governs the genomic access of transcription factors. Across all three compartments, a protein’s diffusive behaviour is not incidental to its function but is itself a direct readout of the physical organisation of its environment, establishing FCS as a uniquely quantitative bridge between molecular dynamics and cellular decision-making.

## 1. Introduction

Eukaryotic cell function is spatially organised: division, differentiation, signal transduction, and genome maintenance each depend on the dynamic localisation of proteins to specific intracellular compartments at defined times [1,2]. Yet these proteins must navigate a succession of fundamentally different physical environments, from the lipid membrane at the cell surface to the chromatin-packed nucleus at its core. The plasma membrane confines proteins to a quasi-2D bilayer ~5 nm thick, where lateral organisation into lipid domains and cytoskeletal compartments determines whether a receptor can cluster, signal, or be internalised [3,4,5,6]. The cytoplasm presents a crowded 3D landscape of organelles, cytoskeletal networks, and macromolecular assemblies that simultaneously obstruct passive diffusion and provide the tracks for active transport [7,8,9,10]. The nucleus compacts metres of genomic DNA into a 10–20 μm diameter volume, creating a chromatin architecture that does not merely house the genome but actively regulates which proteins can reach it, when, and in what form [11,12,13]. Understanding how proteins navigate these environments, how their dynamics are shaped by the physical organisation of each compartment, and how those dynamics in turn execute the cell’s biological decisions, is a central challenge of quantitative cell biology.

Fluorescence correlation spectroscopy (FCS) has emerged as an indispensable tool for meeting this challenge. First introduced by Magde, Elson, and Webb in 1972 [14], FCS extracts quantitative information about molecular diffusion, concentration, and interactions by analysing temporal fluctuations in fluorescence intensity within the small observation volume of a confocal laser scanning microscope (CLSM) [15,16,17]. As fluorescently labelled molecules diffuse into and out of this femtolitre volume, they generate fluctuations whose duration encodes molecular mobility and whose amplitude encodes molecular number [18,19]. The autocorrelation function (ACF), which compares the fluorescence signal with a time-delayed version of itself, recovers the diffusion coefficient and local concentration of the population under observation [18,19]. This approach achieves single-molecule sensitivity while retaining the statistical power of population-level measurements, bridging the gap between single-particle tracking (SPT), which resolves individual trajectories but demands low labelling densities [20,21], and fluorescence recovery after photobleaching (FRAP), which provides population statistics but perturbs the system [22,23]. In its original form, however, FCS was a single-point technique in which a stationary observation volume reported local diffusion through temporal ACF analysis. While transformative for solution studies, this approach is limited in living cells because a single point cannot capture the routes proteins take or how surrounding cellular architecture influences their movement [24,25,26,27]. In a cell, where protein mobility varies with lipid domain composition, cytoskeletal geometry, and chromatin compaction, spatial context is inseparable from biological meaning.

The solution was to introduce a spatial component into the FCS measurement. The first implementation, scanning FCS, moves the confocal illumination volume rapidly along a straight or circular trajectory, applying temporal ACF analysis at each position to map diffusion and binding kinetics across cellular structures [28,29]. Extending this from a temporal ACF to a spatial cross-correlation function (CCF) [30,31], in which fluorescence fluctuations recorded at pixel pairs separated by a set distance along the scan are cross-correlated, gave rise to pair correlation function (pCF) microscopy [31,32]. This method reports the time required for molecules to traverse a defined intracellular distance and maps the long-range diffusive route of a protein population with respect to the barriers it encounters [33]. Transitioning from a 1D line scan to a 2D frame scan by raster scanning the confocal illumination volume across a full field of view enables: (1) temporal image correlation spectroscopy (TICS), which extends temporal ACF analysis across a full frame sequence to spatially map protein mobility with sensitivity to slow dynamics such as DNA-binding interactions [34,35,36]; (2) raster image correlation spectroscopy (RICS), which employs spatial correlation alongside the hidden time structure of the raster scan to recover average diffusion coefficients across the cytoplasm and nucleus [37,38,39,40]; and (3) spatiotemporal image correlation spectroscopy (STICS) [41,42,43], 2D pCF microscopy [44,45], and image mean square displacement (iMSD) [46], which collectively extract maps of molecular flow velocity, transport anisotropy, and the diffusion law governing protein transport [17,24,25]. However, because confocal frame scanning is typically limited to acquisition times on the order of seconds per frame, when implemented on a CLSM, some of these diffusion mapping methods are restricted to slow processes and preclude imaging rapid protein dynamics over larger spatial scales.

**Figure 1:**
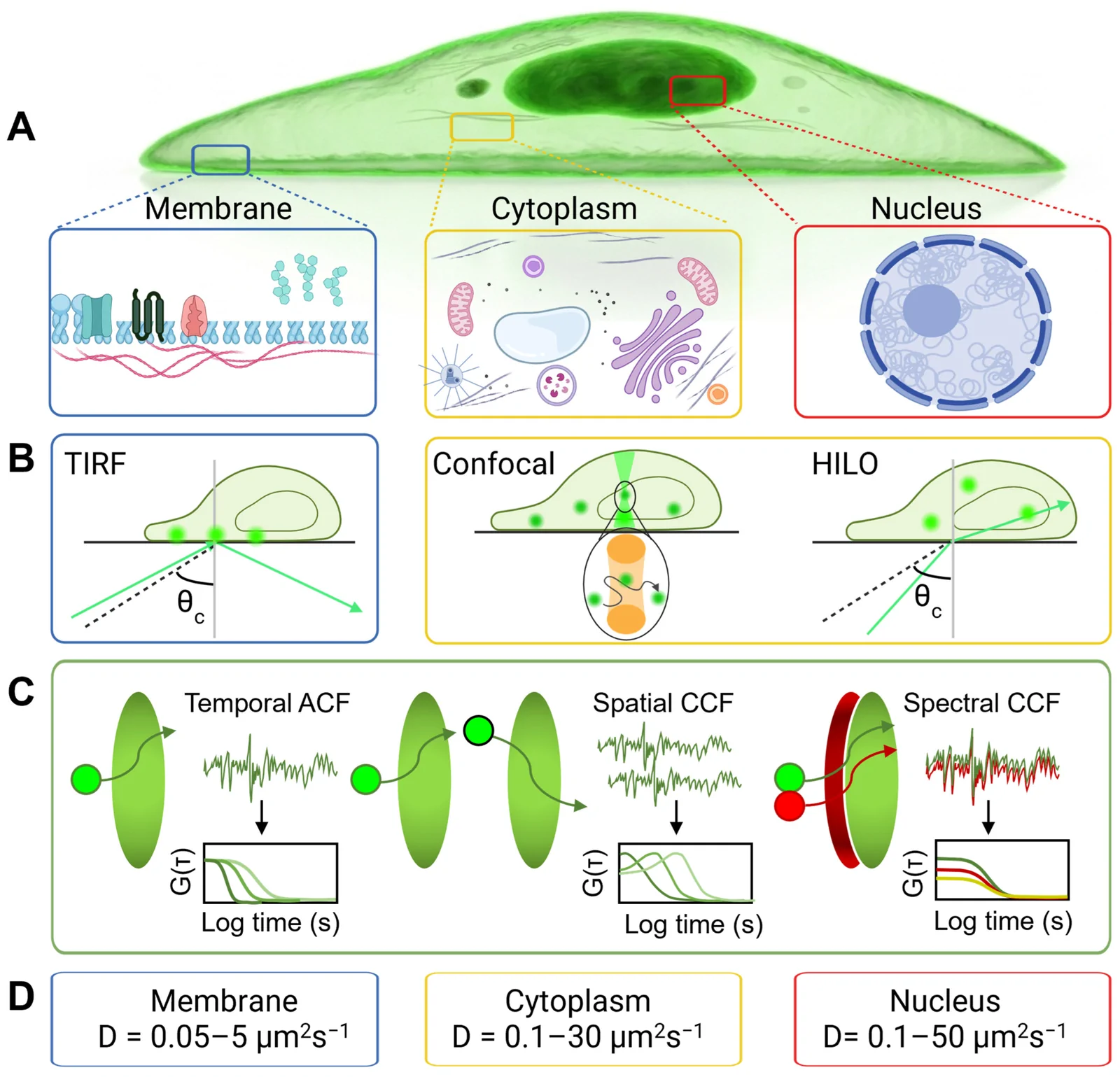
FCS measurement platforms and analytical methods across cellular compartments. (**A**,**B**) Schematic of a eukaryotic cell showing the three physically distinct compartments a protein navigates from the cell surface to its genomic targets: plasma membrane, cytoplasm, and nucleus, with the matched optical platform for each: TIRF for the plasma membrane, and confocal or HILO (or SPIM) for the cytoplasm and nucleus. (**C**,**D**) The three core FCS correlation analyses and expected protein mobility ranges by compartment. Temporal ACF recovers diffusion coefficient and concentration from fluctuations at a single focal volume. Spatial CCF cross-correlates spatially separated pixel pairs to map molecular transport and barriers. Spectral CCF cross-correlates two detection channels to quantify co-diffusion and interactions.

To overcome this limitation, alternative optical platforms have been employed to extend FCS to compartments and timescales inaccessible to confocal illumination. Total internal reflection fluorescence (TIRF) illumination restricts excitation to an evanescent field decaying within ~100 nm of the glass–water interface, a geometry that not only enables frame scan acquisition across thousands of pixels at millisecond sampling, but uniquely selects for the basal plasma membrane, providing a level of membrane specificity that confocal illumination cannot achieve [47,48]. At this temporal resolution and with this spatial selectivity, STICS, 2D pCF microscopy, and iMSD become applicable to membrane protein dynamics, opening access to lipid domain organisation, receptor clustering, and directed membrane flow on spatiotemporal scales inaccessible to confocal frame scanning [49,50,51]. For compartments deeper within the cell, where TIRF cannot penetrate, highly inclined and laminated optical sheet (HILO) microscopy creates a thin oblique illumination sheet of approximately 1–2 μm that penetrates beyond the evanescent field while maintaining substantially better background rejection than widefield illumination [52,53], enabling the same suite of spatiotemporal analyses to be applied to cytoplasmic and nuclear protein dynamics at the acquisition rates they demand. Single-plane illumination microscopy (SPIM) extends this further still, illuminating only the observed plane with a perpendicular light sheet, providing intrinsic optical sectioning, reducing photodamage by orders of magnitude relative to confocal scanning, and enabling simultaneous FCS measurements across thousands of pixels in 3D specimens and intact living organisms [54,55]. Together, TIRF, HILO, and SPIM extend FCS to the full range of cellular environments and dynamics by matching the optical configuration to the physical properties of each compartment (Figure 1A,B).

The mathematical foundations of FCS and its related methods are reviewed elsewhere [16,17]; thus, here we focus on how these approaches have been applied across distinct cellular environments to reveal the biophysical principles governing protein trafficking in living cells. We review FCS methodologies spanning single-point temporal ACF, imaging-based spatiotemporal correlation analyses, multichannel extensions enabling spectral CCF analysis [56,57,58,59,60,61,62,63] and multiplexed analyses beyond fluorescence intensity [64,65,66,67,68,69,70] (Figure 1C,D). Organised from the plasma membrane to the nucleus, we discuss how each cellular environment shapes both experimental approach and the biological questions that can be addressed. At the plasma membrane, the quasi-2D geometry and sub-100 nm organisation governing receptor signalling require TIRF-based illumination with STICS and diffusion law analysis to resolve nanodomain trapping and cytoskeletal compartmentalisation [71,72]. In the cytoplasm, the crowded 3D environment demands confocal and light-sheet approaches combined with RICS and iMSD to distinguish diffusion from directed transport and map where signalling occurs [38,46,73]. In the nucleus, chromatin organisation partitions the interphase volume into accessible and inaccessible domains, requiring confocal and light-sheet approaches with TICS and pair correlation analysis to quantify intranuclear transport and DNA binding dynamics [32,36,74,75]. We also highlight recent advances, including single-photon avalanche diode (SPAD) array detectors [76,77,78] and machine learning-assisted, phasor-based, and Bayesian analytical approaches [79,80,81], that continue to expand the dynamic range of FCS. Collectively, these studies demonstrate that the physical organisation of cellular compartments is an active regulator of protein function, and FCS provides the spatiotemporal resolution needed to measure both simultaneously.

## 2. Plasma Membrane

### 2.1. Overview of the Compartment

**Figure 2:**
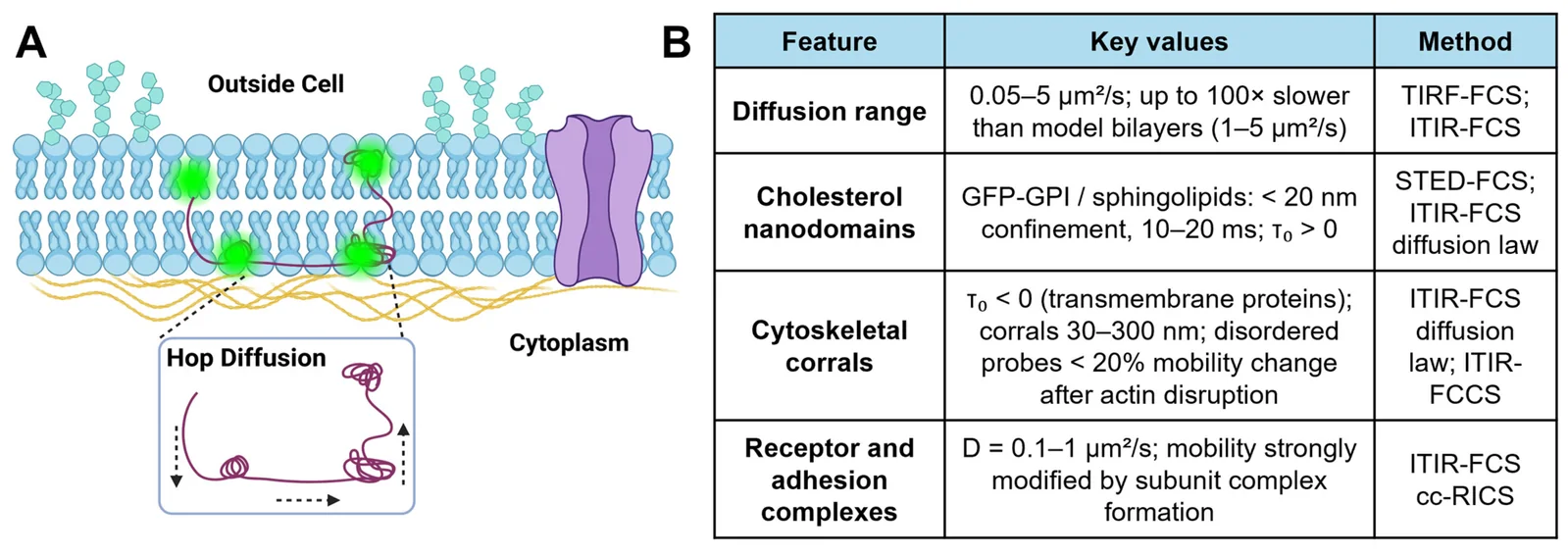
Plasma membrane organisation resolved by FCS. (**A**) Schematic of the plasma membrane. (**B**) Summary of FCS parameters characterising plasma membrane organisation in living cells [71,83,95,101,102,103].

Beyond its role as the cell’s selective barrier and signalling platform, the plasma membrane is a dynamic organisational scaffold whose quasi-2D geometry makes lateral, rather than 3D, organisation the key determinant of receptor clustering, signalling, and internalisation [3,4,82]. The membrane exhibits complex lateral organisation across multiple spatial scales: cholesterol-enriched lipid nanodomains smaller than 20 nm [71], cytoskeletal corrals of 30–300 nm imposed by the underlying actin meshwork [83,84,85], and larger-scale signalling platforms and endocytic structures at the microscale [34,35,42]. Together, these features create a highly heterogeneous diffusion landscape in which a protein’s mobility and domain association determine its functional output. High protein surface densities (20,000–50,000 per μm^2^) [86,87] and reorganisation on millisecond-to-minute timescales [3,88] further characterise the membrane as a temporally dynamic compartment (Figure 2A).

### 2.2. Preferred Optical and FCS Approaches

TIRF is the natural platform for membrane FCS, selectively exciting fluorophores at the basal membrane while eliminating cytosolic background, a compartment specificity unmatched by confocal illumination [47,48]. In its simplest implementation, single-point TIRF-FCS employs a temporal ACF analysis to recover local lateral diffusion coefficients and molecular concentrations [89,90,91]. Camera-based imaging FCS or imaging total internal reflection FCS (ITIR-FCS) extends temporal ACF analysis across the entire field of view, generating pixel-by-pixel maps of membrane diffusion and concentration at millisecond frame rates [47,48]. This approach is closely related to TICS [34,35,42], while two-colour implementations using a spectral CCF quantify co-diffusion and protein–protein interactions [56,92,93]. Moving beyond temporal correlation, STICS generates pixel-by-pixel velocity maps of membrane flow and directionality from successive image frames, enabling directed transport to be distinguished from lateral diffusion [41,94]. The related iMSD approach extracts membrane diffusion laws from the temporal broadening of the spatial correlation function, distinguishing free, confined, and hop diffusion [46,51]. Complementing these methods, the spatial CCF equivalent of ITIR-FCS (ITIR-FCCS) and 2D pCF compute spatial cross-correlations between neighbouring pixels to map transport anisotropy without prior knowledge of transport direction [45,83].

The FCS diffusion law provides an orthogonal measure of membrane organisation by plotting the apparent diffusion time (τ_D_) as a function of observation area (ω^2^), where the y-intercept (τ_0_) reports deviations from free Brownian diffusion arising from sub-resolution confinement or barriers [72,95]. Positive τ_0_ values report transient trapping within lipid nanodomains, whereas negative τ_0_ values indicate hop diffusion between cytoskeletal corrals, with imaging FCS, ITIR-FCS, and ITIR-FCCS extending this framework to more complex membrane-binding behaviours [95,96]. Finally, specialised FCS variants further expand the accessible spatial scales: z-scan FCS corrects focal-position artefacts in lipid bilayers [97], stimulated emission depletion FCS (STED-FCS) reduces the observation spot diameter to ~40 nm to resolve cholesterol-dependent nanodomains [98,99], and fluorescence lifetime correlation spectroscopy (FLCS) exploits membrane environment-dependent fluorophore lifetimes to reveal otherwise inaccessible features of membrane structure [100]. Together, these complementary approaches quantify membrane organisation from nanometre-scale lipid domains to whole-cell membrane dynamics. The major membrane diffusion modes, the FCS methods used to measure them, representative mobility ranges, and their biological insights are summarised in Figure 2B.

### 2.3. Quantitative Mobility Characteristics and Insights from Inert Tracers

Model membrane systems such as supported lipid bilayers (SLBs) and giant unilamellar vesicles (GUVs) establish the unconstrained baseline of cholesterol-driven liquid-ordered versus liquid-disordered phase coexistence and free 2D Brownian diffusion, with diffusion coefficients typically ranging from 1 to 5 μm^2^/s [104,105]. In contrast, diffusion in live-cell membranes spans a far wider range and can be up to 100-fold slower [106], reflecting a level of membrane organisation that extends beyond simple lipid viscosity. More specifically, plasma membrane proteins adopt diffusion coefficients that span 0.05–5 μm^2^/s depending on their local membrane microenvironment [72,83,103]. Freely diffusing lipid probes such as DiI occupy the upper end of this range (1–3 μm^2^/s), while GPI-anchored proteins associated with cholesterol-rich nanodomains typically diffuse at 0.1–1 μm^2^/s, and transmembrane proteins coupled to the actin cytoskeleton are further slowed to 0.05–0.5 μm^2^/s [72,83,84,101,103]. These distinct diffusion coefficients arise from different physical modes of membrane transport, which can be distinguished using FCS diffusion law and spatial CCF analysis. Diffusion law analysis resolves free Brownian diffusion (τ_0_ = 0) from transient nanodomain trapping (τ_0_ > 0) and cytoskeleton-mediated hop diffusion (τ_0_ < 0), with spatial CCF analysis additionally able to measure the directionality of these dynamics [72,95,96]. Fluorescent lipid analogues and minimally interacting membrane probes establish the experimental basis for these diffusion regimes. DiI, a liquid-disordered phase marker, diffuses at 2.5 ± 2.0 μm^2^/s in the basal plasma membrane of adherent SH-SY5Y neuroblastoma cells, with its large spatial variability reflecting intrinsic membrane heterogeneity [83]. Cholesterol depletion with methyl-β-cyclodextrin (MβCD) or actin disruption with latrunculin A increases DiI mobility by less than 20%, indicating that liquid-disordered probes are relatively insensitive to either cholesterol-rich nanodomains or the cortical cytoskeleton [83]. In contrast, glycosylphosphatidylinositol-anchored green fluorescent protein (GFP-GPI) exhibits a strongly positive diffusion law intercept, consistent with transient confinement within ~20 nm cholesterol-dependent nanodomains for 10–20 ms, whereas DiI-C18 shows an intercept near zero, confirming free diffusion across all observation scales [71,106]. STED-FCS further demonstrated that confinement of GPI-anchored proteins and sphingomyelin is largely abolished in actin-free plasma membrane vesicles, whereas ganglioside GM1 retains partial confinement, indicating distinct contributions from cholesterol- and cytoskeleton-dependent mechanisms [103]. These organisational differences are largely invisible to single-point FCS, which reports similar average diffusion coefficients despite fundamentally different underlying transport mechanisms, and highlight why diffusion law analysis is essential for correctly diagnosing membrane organisation. Perturbation experiments further confirmed these relationships: the sphingolipid-binding domain (SBD) raft probe becomes approximately 4-fold more mobile following actin disruption, temperature-dependent measurements reveal partial melting of cholesterol nanodomains, and CCF analysis following MβCD treatment quantifies the transient disruption and subsequent recovery of membrane organisation [83,106].

### 2.4. Key Biological Applications and Insights

The application of FCS to plasma membranes has transformed understanding of membrane organisation across multiple spatial scales. Landmark STED-FCS studies demonstrated that sphingolipids and GPI-anchored proteins are transiently confined for 10–20 ms within cholesterol-dependent complexes smaller than 20 nm, whereas phosphoglycerolipids diffuse freely across all spatial scales [71]. Scanning STED-FCS further showed that this transient trapping cannot be explained solely by classical lipid phase separation, implicating alternative cholesterol-mediated molecular interactions in membrane organisation [99,107], while detailed experimental protocols have since enabled these measurements across diverse membrane systems [108]. Complementary ITIR-FCCS studies demonstrated that membrane heterogeneity is organised on at least two spatial scales, with sub-resolution cholesterol-dependent nanodomains superimposed on larger-scale cytoskeletal compartments [83,101]. More recently, systematic analysis has shown that membrane-binding processes and certain lipid species require extensions to the classical interpretation of the FCS diffusion law [96]. Beyond membrane organisation, fluorescence fluctuation methods have provided quantitative insight into how receptor and adhesion complex dynamics regulate signalling. ITIR-FCS coupled with diffusion-law analysis demonstrated that the epidermal growth factor receptor (EGFR), which diffuses at 0.1–0.3 μm^2^/s, undergoes stimulation-dependent interactions with both cholesterol-rich membrane domains and the actin cytoskeleton [101,102]. Cross-correlation RICS (cc-RICS) combined with number and brightness (NB) analysis resolved the assembly and disassembly of integrin-associated adhesion complexes, revealing phosphorylation-dependent paxillin–focal adhesion kinase (FAK) interactions, with the paxillin–FAK complex diffusing at 0.5–1 μm^2^/s during focal adhesion maturation [58,109,110,111].

FCS has also provided mechanistic insight into membrane disruption by pathogens and cytotoxic peptides. Scanning FCS alongside spectral CCF analysis demonstrated that the influenza A virus M2 ion channel recruits matrix protein M1 to the plasma membrane at viral budding sites [112]. Combined confocal FCS and ITIR-FCS showed that the antimicrobial peptide aurein 1.2 reduces lipid diffusion coefficients by 50–90% and induces a positive diffusion law intercept, indicating peptide-driven membrane domain formation preceding disruption [113]. Bayesian TIRF-FCS further revealed that monomeric human islet amyloid polypeptide (hIAPP) nucleates membrane domains in pancreatic β-cells at sub-micromolar concentrations, destabilising membranes through a carpet mechanism before detectable aggregation occurs [114,115]. Mapped onto the hierarchy introduced in Section 2.1, these findings show that nanoscale cholesterol-dependent nanodomains transiently trap GPI-anchored proteins and sphingolipids [71,83,103], facilitating receptor–effector interactions. At the mesoscale, actin corrals restrict transmembrane protein mobility through hop diffusion [84,101]. At the microscale, signalling platforms, protein clusters, and endocytic structures generate functionally specialised membrane regions [34,42]. Continued integration of STED with imaging FCS will enable direct visualisation of the nanodomains inferred from diffusion-law analysis, providing new mechanistic insight into plasma membrane organisation [49,71,108].

## 3. Cytoplasm

### 3.1. Overview of the Compartment

**Figure 3:**
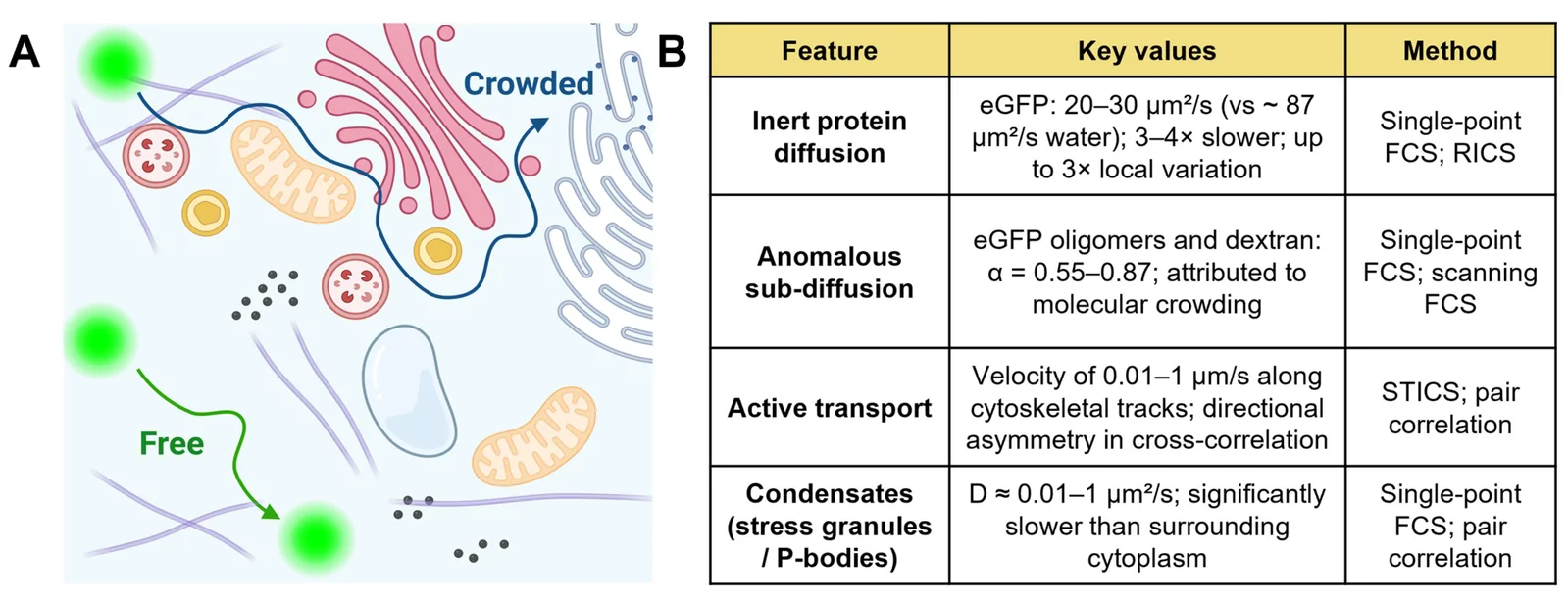
Cytoplasmic organisation resolved by FCS. (**A**) Schematic of the cell cytoplasm. (**B**) Summary of FCS parameters characterising cytoplasmic organisation in living cells [8,37,119,120,125,126,130].

The cytoplasm presents a fundamentally different environment from the plasma membrane, transitioning from the quasi-2D constraint of the bilayer to a crowded 3D landscape through which proteins must navigate a dense, dynamic network of membrane-bound organelles, including the endoplasmic reticulum, mitochondria, endosomes, and transport vesicles, interwoven with actin and microtubule cytoskeletal filaments [10]. Total intracellular protein concentrations reach 50–400 mg/mL, occupying 20–40% of the cytoplasmic volume and producing an effective viscosity approximately 3–4-fold greater than that of water [7,8]. Consequently, proteins rarely undergo simple Brownian diffusion. Instead, macromolecular crowding and transient interactions produce anomalous sub-diffusion, while cytoskeletal networks support directed intracellular transport of cargo [8,116,117,118,119]. Unlike the plasma membrane, where lateral diffusion dominates, the cytoplasm supports continual interplay between passive diffusion and active transport, with their relative contributions varying with protein identity, cargo size, and physiological state. This coexistence makes the cytoplasm the principal environment in which FCS methods must distinguish diffusion from transport while quantifying how crowding shapes molecular mobility (Figure 3A).

### 3.2. Preferred Optical and FCS Approaches

Single-point FCS on a CLSM quantifies diffusion coefficients and binding kinetics at defined cytoplasmic locations with microsecond-to-millisecond temporal resolution, sufficient to capture rapid soluble-protein diffusion [8,120,121,122]. RICS exploits the raster-scan time structure to recover an average diffusion coefficient per square region of interest (ROI), separating freely diffusing from slow or transiently bound species via their distinct correlation signatures; ROI-to-ROI comparison reveals mobility heterogeneity near organelles and the perinuclear environment [37,38,40]. Arbitrary-region RICS (ARICS) further extends this approach to user-defined ROI shapes, enabling diffusion measurements within irregularly shaped cytoplasmic subregions while excluding organelles and other immobile structures [123,124]. Scanning FCS extends this to pixel-level spatial resolution by recovering a diffusion coefficient at each position along a defined line scan, directly correlating local mobility with cytoskeletal structures and organellar boundaries [74,125]. Where transport is facilitated or restricted by intracellular architecture, 1D line-scan pCF microscopy measures the time required for molecules to traverse defined intracellular distances, while asymmetry between forward and reverse cross-correlations reports the preferred direction of transport with respect to actin and microtubule organisation [64,126,127].

Complementing these approaches, STICS extracts pixel-by-pixel velocity vectors from frame-scan data, generating maps of cytoplasmic flow fields that distinguish directed motor-driven transport from passive diffusion [118,119], while the related iMSD approach analyses changes in the spatial correlation function over time to distinguish free Brownian diffusion, anomalous sub-diffusion, and active transport within the crowded cytoplasm, with an anomalous exponent (α) below 1 reporting crowding-induced slowing [46]. Because the cytoplasm contains both freely diffusing proteins and large moving organelles, STICS and iMSD incorporate immobile-fraction subtraction and temporal detrending to suppress slow intensity fluctuations before extracting transport dynamics [46,116]. k-Space image correlation spectroscopy (kICS) offers a complementary approach by analysing images in the frequency domain rather than real space, which has the practical advantage of separating genuine molecular transport from fluorophore blinking or photobleaching artefacts that can otherwise bias diffusion measurements [128,129]. For faster acquisition than confocal scanning permits, HILO and SPIM-based imaging enable camera-based imaging FCS to resolve rapid diffusion and transport in the crowded cytoplasm [52,53]. For example, TICS-like analysis across a SPIM data acquisition enables volumetric diffusion mapping with reduced photobleaching during dynamic processes such as cell division and embryogenesis [54,55]. Finally, SPAD array detectors enable parallel single-point FCS measurements at multiple cytoplasmic locations with nanosecond temporal resolution, providing access to fast and directed-diffusive dynamics [76,77]. Collectively, these FCS-based approaches distinguish passive diffusion, directed transport, transient binding, and barrier crossing throughout the cytoplasm, with representative readouts summarised in Figure 3B.

### 3.3. Quantitative Mobility Characteristics and Insights from Inert Tracers

Inert fluorescent proteins establish the baseline against which the effects of cytoplasmic crowding and active transport can be understood. Cytoplasmic protein diffusion coefficients span approximately 0.1–30 μm^2^/s, reflecting the diverse physical environments encountered by proteins within this compartment [44,46,74,131]. Small fluorescent proteins such as monomeric enhanced green fluorescent protein (eGFP) diffuse at 20–30 μm^2^/s in the cytoplasm when measured by single-point FCS, approximately 3–4-fold slower than in aqueous solution (~87 μm^2^/s), demonstrating the combined effects of macromolecular crowding and transient non-specific interactions [125]. Consistent with these measurements, RICS also recovers eGFP diffusion coefficients of approximately 25 μm^2^/s across the cytoplasm, but with up to 3-fold local variation near organelles and perinuclear regions within a single image stack [37,111]. Scanning FCS measurements using eGFP and mCherry similarly identified a viscosity-related retardation factor of approximately 3–4 relative to water across multiple cell lines [92,125,132]. Extending these measurements into higher-dimensional imaging approaches, SPIM-FCS resolved the coexistence of a rapidly diffusing eGFP population (D = 35 ± 8 μm^2^/s; ~85% of molecules) and a slower, confined population (D = 0.4 ± 0.3 μm^2^/s; ~15%), demonstrating that even inert tracers experience transient confinement within the cytoplasmic environment [92,133].

Single-point FCS measurements using dextrans of increasing molecular size revealed that cytoplasmic crowding drives a transition from Brownian to anomalous diffusion, with α decreasing to approximately 0.55–0.87 for larger tracers, indicating enhanced confinement by intracellular structures [8,87]. Importantly, however, these constraints do not prevent larger molecules from accessing and moving through the cytoplasm. Rather, 1D and 2D pCF microscopy demonstrated that eGFP oligomers spanning different sizes can simultaneously traverse the cytoplasm over defined spatial distances, indicating that the cytoplasm remains broadly accessible despite increased steric hindrance [68,74]. Polarisation-resolved FCS further separated different contributions to crowding by showing that translational diffusion of inert tracers is reduced approximately 3-fold relative to dilute buffer, whereas rotational diffusion is affected only 2-fold, distinguishing molecular confinement from simple increases in bulk viscosity [134]. More recently, integration of FCS with fluorescence lifetime imaging enabled monitoring of cytoplasmic crowding during cellular stress, revealing rapid reductions in eGFP mobility associated with hyperosmotic compression [135].

### 3.4. Key Biological Applications and Insights

These FCS-based measurements of cytoplasmic diffusion provide a quantitative foundation for understanding how this compartment regulates and exploits changes in molecular mobility to control biological processes. For example, spatiotemporal variants of FCS have shown that the cytoplasm accommodates directed transport of specific cargo in parallel with free diffusion. In particular, STICS and kICS have generated spatial maps of cytoplasmic flow that reveal distinct transport domains associated with different cytoskeletal tracks and cargo velocities of 0.01–1 μm/s [118,119], while pCF microscopy resolves the direction of that transport, with asymmetry in the forward and backward cross-correlation functions reporting the net directionality of Rho GTPase and F-actin bundle flow with respect to the direction of cell migration and along three-dimensional cell protrusions [64,126]. RICS showed that paxillin-eGFP diffuses freely throughout the cytoplasm but becomes locally immobilised upon binding FAK complexes at focal adhesions, directly mapping the transition from freely diffusing to adhesion-engaged populations within a single acquisition [37,58], while a SPIM-based spectral CCF analysis simultaneously monitored c-Fos–eGFP and c-Jun–monomeric red fluorescent protein (mRFP) heterodimerisation, demonstrating that heterodimer formation begins in the cytoplasm before preferential nuclear accumulation [92,133].

More recently, FCS has become an important tool for studying diffusion within both membraneless and membrane-bound cytoplasmic compartments. Within cytoplasmic liquid–liquid phase-separated condensates, including stress granules and processing bodies, single-point FCS has revealed that proteins exhibit substantially reduced diffusion coefficients of approximately 0.01–1 μm^2^/s in these environments compared with the surrounding cytoplasm, while pCF microscopy has enabled quantification of molecular exchange across condensate boundaries [130]. Similarly, RICS analysis of internalised albumin bound to its recycling receptor, the neonatal Fc receptor (FcRn), within endosomal compartments revealed restricted mobility (D = 0.04–0.12 μm^2^/s), directly linking receptor engagement to the diffusive behaviour and trafficking fate of cargo during endosomal recycling [136]. Taken together, these complementary FCS approaches establish that the defining feature of the cytoplasm is the coexistence of crowding-limited diffusion, active motor-driven transport, and membraneless and membrane-bound compartments within which mobility is further restricted. Looking forward, advances in computational FCS analysis, including Bayesian model selection [81] and neural network-based parameter extraction from shorter, noisier datasets [79], promise to improve both FCS data acquisition and interpretation, enabling more robust quantification of molecular transport within the heterogeneous, multiscale environment of the cytoplasm.

## 4. Nucleus

### 4.1. Overview of the Compartment

**Figure 4:**
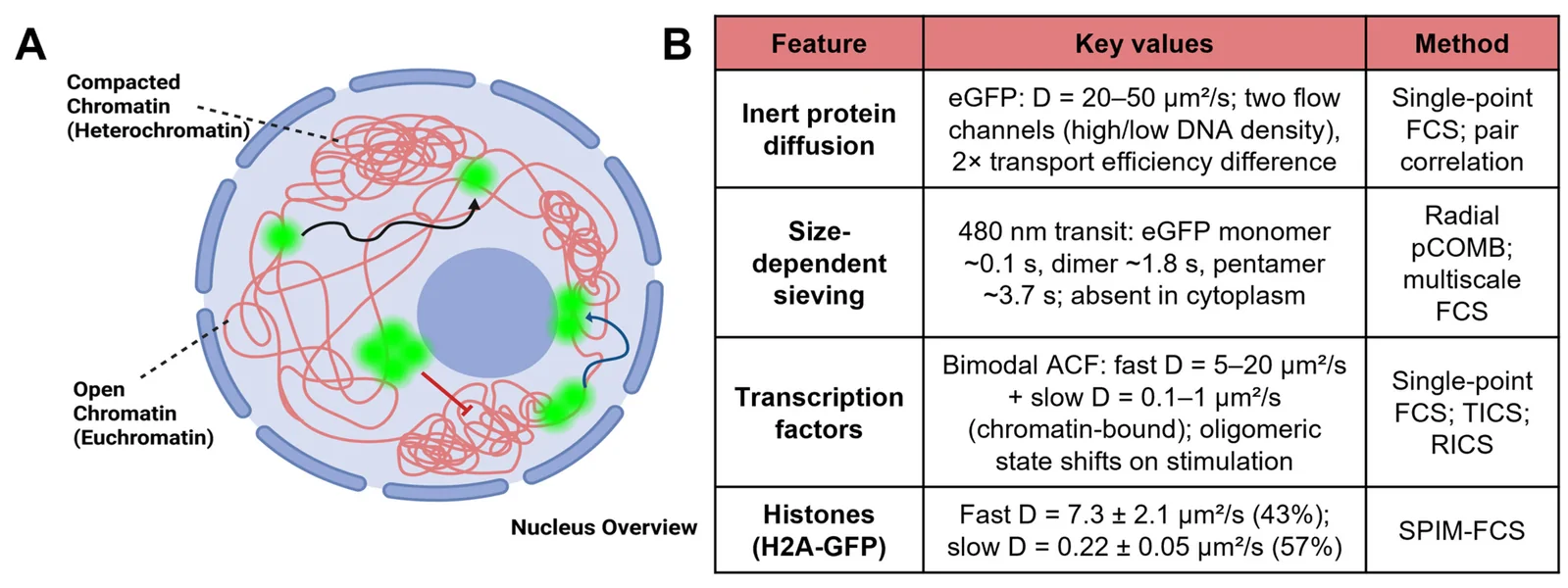
Nuclear organisation resolved by FCS. (**A**) Schematic of the cell nucleus. (**B**) Summary of FCS parameters characterising nuclear organisation in living cells [32,36,68,74,92,142,147].

Like the plasma membrane and cytoplasm, the nucleus presents a structurally complex and highly heterogeneous trafficking environment in which proteins must navigate a dense chromatin landscape to locate specific genomic targets [13]. Approximately 2 m of genomic DNA is compacted into a nucleus of ~10–20 μm diameter, forming a hierarchical chromatin network that partitions the nucleoplasm into regions of distinct accessibility [137,138]. This organisation generates a spatially heterogeneous diffusion landscape in which the accessible volume varies between transcriptionally active euchromatin and transcriptionally repressive heterochromatin, while continuous chromatin remodelling dynamically reshapes these pathways over time [139,140]. Exchange between the cytoplasm and nucleus is regulated by nuclear pore complexes (NPCs) [127,141], while the chromatin mesh itself introduces a physical sieve whose pore size is comparable to the hydrodynamic radii of many soluble proteins, imposing strong size- and interaction-dependent constraints on diffusion that are not observed in the cytoplasm [68,74]. Together, these features define the nucleus as a selective, dynamically reconfigured diffusion environment in which protein mobility is governed by crowding, chromatin accessibility, binding interactions, and organisation across length scales, all further modulated through the cell cycle as chromatin compaction and DNA replication reshape the effective diffusion landscape (Figure 4A).

### 4.2. Preferred Optical and FCS Approaches

Single-point confocal FCS has been used extensively to measure nuclear protein dynamics, resolving fast freely diffusing species (D ~ 5–50 μm^2^/s) alongside slow chromatin-bound populations (D ~ 0.1–1 μm^2^/s) within a single bimodal ACF [142,143,144]. Two-component fitting of the nuclear ACF separates these fast and slow populations and quantifies their fractional contributions, providing a direct readout of the fraction of a transcription factor engaged with chromatin at any moment [144]. Where binding kinetics do not partition cleanly into two states, power-law fitting of the ACF instead captures a continuum of chromatin residence times, better describing the heavy-tailed dwell-time distributions observed for many transcription factors [145]. High-throughput FCS workflows have now extended this to proteome-scale measurements, classifying 53 nuclear proteins by their chromatin binding and complex formation dynamics from 60,000 measurements in 10,000 living human cells [146]. For spatially resolved measurements, confocal frame-scan acquisitions support two complementary analyses: TICS extends temporal ACF analysis across a full frame sequence, generating pixel-by-pixel maps of nuclear protein mobility with particular sensitivity to slow DNA-binding interactions [36], while RICS exploits the hidden time structure of the raster scan to recover an average diffusion coefficient across the nucleus with full spatial context from a single image stack [147,148]. Scanning FCS extends single-point measurements along a defined path, mapping local diffusion coefficients as a function of position across the nucleus [74,120], while intensity-sorted FCS (IS-FCS) extends this by simultaneously acquiring a chromatin density marker alongside the protein of interest, enabling ACF analysis to be sorted by local DNA density and revealing subtle diffusion differences between euchromatin and heterochromatin environments [149].

Applied to the same 1D line scan, pCF analysis reveals how chromatin architecture channels molecular flow and enables the directionality of molecular movement across regions of high and low DNA density to be detected [32,33,150,151]. The two-colour cross-pCF variant acquires line scans in two spectral channels simultaneously, tracking transport as a function of heterotypic interaction between spectrally distinct fluorescent species and enabling dimer-dependent nuclear trafficking to be directly dissected [61,63,64,152]. Combining pCF analysis of fluorescence fluctuations with moment-based brightness analysis of oligomeric state, a technique termed pair correlation of molecular brightness (pCOMB), enables oligomer-dependent nuclear trafficking to be detected [65,68]. This latter type of analysis is particularly suited to nuclear transcription factors that exist at low copy numbers and whose oligomeric state transitions are the key regulatory event governing their genomic accessibility [147,148,153,154]. For broader-field acquisitions, HILO and SPIM illumination penetrate beyond the basal membrane to reach the nucleus while maintaining substantially better background rejection than widefield illumination, making them well suited to camera-based imaging FCS of nuclear proteins across the entire nucleus [92]. Together, these approaches provide complementary measurements of diffusion, chromatin interactions, and intranuclear transport. Representative mobility regimes and FCS-derived biological insights are outlined in Figure 4B.

### 4.3. Quantitative Mobility Characteristics and Insights from Inert Tracers

Inert fluorescent tracers that diffuse through the nucleus without sequence-specific DNA binding have been foundational for understanding how chromatin architecture regulates molecular traffic [32,68,74,142,143]. Single-point FCS measurements across different nuclear regions found that small inert proteins such as monomeric eGFP exhibit diffusion coefficients of 10–50 μm^2^/s that were not necessarily correlated with local chromatin density, indicating that these molecules can navigate the nuclear space rapidly, but by a means that local chromatin structure alone does not predict [142]. pCF microscopy resolved this apparent discrepancy by revealing that the interphase nucleus contains spatially organised transport pathways, with molecular flow occurring through distinct channels associated with regions of higher and lower DNA density [32]. Transport efficiency differed approximately 2-fold between these pathways, demonstrating that chromatin organisation regulates not only the amount of available space but also the connectivity of diffusion routes [150]. Transient communication events between these channels occurred on ~300 ms timescales and were abolished during mitotic chromosome condensation, demonstrating that chromatin organisation is dynamically regulated as a function of the cell cycle [151]. The size dependence of nuclear transport was further revealed using inert eGFP oligomers as molecular probes. Multiscale FCS measurements of eGFP monomers, dimers, and larger oligomers, together with radial pCOMB analysis, demonstrated that increasing tracer size progressively reduces mobility and alters spatial accessibility within the nucleus [68,74]. Larger oligomers become excluded from specific chromatin environments while being preferentially retained within others, demonstrating that the nucleus imposes a size-dependent molecular sieving effect. pCOMB directly quantified this size-dependent retardation, with eGFP traversing 480 nm nuclear distances in approximately 0.1 s, compared with approximately 1.8 s for eGFP dimers and 3.7 s for eGFP pentamers [68]. Importantly, identical measurements in the cytoplasm showed minimal size-dependent effects, establishing that this molecular sieving behaviour arises from the structured chromatin landscape rather than general intracellular crowding.

### 4.4. Key Biological Applications and Insights

The application of FCS-based methods to the cell nucleus has transformed understanding of how DNA-binding proteins navigate chromatin to locate genomic targets [155]. Scanning FCS has shown that transcription factors display bimodal mobility distributions, with fast components at 5–20 μm^2^/s representing chromatin-scanning populations and slow fractions at 0.1–1 μm^2^/s representing site-engaged populations [68,74]. At the proteome scale, high-throughput single-point confocal FCS has classified dozens of nuclear proteins by their chromatin-binding and complex-formation dynamics across tens of thousands of living cells [146]. Stably chromatin-associated proteins such as histones exhibit predominantly slow diffusion: SPIM-FCS measurements of H2A-GFP resolved a fast component (D = 7.3 ± 2.1 μm^2^/s, 43%) and a slow chromatin-associated component (D = 0.22 ± 0.05 μm^2^/s, 57%), directly quantifying the fraction of histones dynamically exchanging from chromatin [92]. Beyond measuring mobility, FCS approaches have revealed how molecular interactions and oligomeric state regulate nuclear function. Coupling brightness analysis with FCS has revealed that the glucocorticoid receptor (GR) transitions from dimer to tetramer upon DNA binding, a switch critical to transcriptional activation [153,154]. Subsequent FCS work also showed that, as in the cytoplasm, where liquid–liquid phase separation generates membraneless compartments amenable to FCS analysis [130], GR can assemble into dynamic nuclear condensates, linking receptor organisation and molecular mobility to regulation of transcriptional output [156,157]. Oligomeric assembly has likewise been resolved for other nuclear factors, with RICS and cc-RICS used to resolve the assembly of nuclear transcription factor Y (NF-Y) into a pre-formed NF-YB–NF-YC heterodimer prior to NF-YA binding [147], as well as show that SRY-box transcription factor 18 (SOX18) homodimerisation drives high-affinity chromatin binding, with dominant-negative mutants simultaneously perturbing mobility, oligomeric state, and chromatin affinity in a manner that mechanistically explains severe vascular phenotypes [158].

FCS-based correlation approaches have also revealed how nuclear architecture regulates protein targeting and genome surveillance. Two-colour cross-pCF analysis directly tracked recruitment of p53 binding protein 1 (53BP1) to laser-induced DNA double-strand breaks, demonstrating a capture-and-lock mechanism governed by chromatin modifications and histone signalling [152]. Similarly, radial pCOMB analysis of signal transducer and activator of transcription 3 (STAT3) revealed anisotropic, dimer-dependent translocation through the nuclear environment, demonstrating that oligomeric state influences access to genomic targets [68]. These correlation approaches have also been extended beyond chromatin to define transport at the nuclear boundary. pCF microscopy has directly visualised the directional asymmetry between active nuclear import and passive export across NPCs [127,141,159], while the triple correlation equivalent of pCF, spatial triple correlation spectroscopy (S3CS), has enabled the sequential binding and release steps of nuclear localisation signal (NLS)-dependent import mediated by importin α and β to be measured [60]. Together, these studies establish that chromatin is not simply a passive scaffold but an active regulator of nuclear transport, where molecular size, oligomerisation, and binding state determine protein accessibility. Looking forward, SPAD-array detectors will further advance FCS-based interrogation of the cell nucleus by enabling detection of molecular flow on faster timescales [77], while label-free interferometric scattering correlation spectroscopy (iSCORS) provides complementary measurements of chromatin condensation dynamics in unlabelled nuclei on the millisecond timescale [160].

## 5. Conclusions

The three compartments surveyed in this review—plasma membrane, cytoplasm, and nucleus—impose fundamentally different physical constraints on protein motion, yet FCS-based approaches reveal a common organisational principle across all three: each environment is structured on spatial and temporal scales that are invisible to conventional imaging but directly accessible through correlation-based analyses. At the membrane, there is sub-diffraction partitioning, which diffusion law and STED-FCS resolve as nanodomains and cytoskeletal corrals tens of nanometres in size. In the cytoplasm, there is the competition between passive, crowding-restricted diffusion and active, motor-driven transport, disentangled by different types of spatiotemporal correlation functions, including RICS, STICS, kICS, and iMSD, each of which independently recovers transport dynamics from a distinct correlation signature. In the nucleus, there is the oligomeric-state-dependent gating imposed by the chromatin meshwork itself, revealed through radial pair correlation of molecular brightness as a sieve that converts a protein’s assembly state into a direct determinant of which genomic regions it can reach. Collectively, these studies demonstrate that FCS transforms diffusive behaviour into a sensitive reporter of cellular organisation. For example, positive or negative intercepts in membrane diffusion law analysis identify the mechanisms underlying receptor confinement before signalling begins, while arrival-time asymmetry in a nuclear–cytoplasmic pCF analysis reveals the directionality of nucleocytoplasmic transport. In this sense, FCS measures far more than where and how fast molecules move; it reveals the physical constraints that organise intracellular processes and, ultimately, determine cellular function.

Several converging technical advances are poised to extend these capabilities further. SPAD array detectors bridge the gap between camera-based imaging FCS and conventional single-point measurements by combining parallel detection with nanosecond temporal resolution across multiple observation volumes. Machine learning-assisted fitting is reducing the data and time required for robust diffusion-coefficient extraction, opening FCS to faster processes than were previously tractable. Phasor-based and Bayesian analysis frameworks are making model-free interpretation of heterogeneous, multi-component diffusion routine rather than exceptional. kICS and its extensions provide a complementary route to recover transport dynamics and number densities from image time series that is robust to complex fluorophore photophysics, extending the analytical toolkit beyond the limits of real-space correlation methods. And label-free approaches such as iSCORS point toward a future in which the dynamic organisation of unlabelled cellular structures can be mapped with the same quantitative rigour currently reserved for fluorescently tagged proteins. Thus, looking ahead, the central challenge is as much integrative as methodological. As FCS-based platforms continue to combine spatial, spectral, and alternate fluorescence parameters within a single acquisition, and as they extend from cultured cells into intact organisms, the prospect is a continuous, quantitative map of protein trafficking from the moment a receptor engages a signal at the plasma membrane to the moment a transcription factor finds its target within the genome, closing the loop between molecular mobility and the biological decisions that mobility makes possible.

## Data Availability

No new data were created or analysed in this study. Data sharing is not applicable to this article.
